# Light People: Prof. John Bowers spoke about silicon photonics

**DOI:** 10.1038/s41377-022-01040-y

**Published:** 2022-12-09

**Authors:** Chenzi Guo, Yating Wan

**Affiliations:** 1 Changchun Institute of Optics, Fine Mechanics and Physics, Chinese Academy of Sciences, Changchun, China; 2 Integrated Photonics lab, King Abdullah University of Science and Technology, Thuwal, Makkah Province Saudi Arabia

**Keywords:** Semiconductor lasers, Optoelectronic devices and components

## Abstract

Silicon photonics is advancing rapidly with many scientific and engineering advances and many new applications for photonics. To highlight the topic, *Light: Science & Applications* invited John Bowers, director of the Institute for Energy Efficiency and distinguished professor from the University of California, Santa Barbara, to talk about the fundamentals and industries, and give a future perspective of silicon photonics. The below is summarized from the video interview of Prof. Bowers. The original interview can be accessed in Supplementary video.

**Figure:**
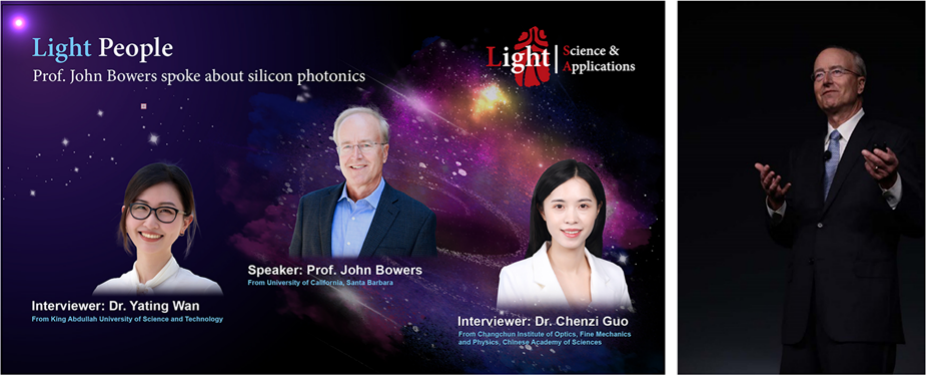
Interviewers and Interviewee

**Short Bio of Prof. John Bowers**

John E. Bowers holds the Fred Kavli Chair in Nanotechnology and is the Director of the Institute for Energy Efficiency and a Distinguished Professor in the Department of Electrical and Computer Engineering of University of California, Santa Barbara. Dr. Bowers received his M.S. and Ph.D. degrees from Stanford University and worked for AT&T Bell Laboratories and Honeywell before joining UC Santa Barbara.

Dr. Bowers is a member of the National Academy of Engineering, the National Academy of Inventors, and a fellow of the IEEE, OSA, and the American Physical Society. He is a recipient of the IEEE Photonics Award, the OSA/IEEE Tyndall Award, the OSA Holonyak Award, the IEEE LEOS William Streifer Award, and the South Coast Business and Technology Pioneer and Entrepreneur of the Year Awards. He is a cofounder of Aurrion, Aerius Photonics, and Calient Networks. He has published two books, 15 book chapters, 900 journal papers, 1200 conference papers, and has received 75 patents. He and his coworkers received the EE Times Annual Creativity in Electronics (ACE) Award for Most Promising Technology for the hybrid silicon laser in 2007.

**Q1: With the exponential growth of communication capacity, will future integrated circuits (ICs) resolve the Input/Output bottleneck?**

A1: The big demands of data centers, particularly for artificial intelligence and machine learning, will continue to drive the need for faster applications and computations in the next decade. 3 nm chips will be in volume next year by TSMC for apple iPhone and Macs. 2 nm chips should be available in 2025. With the traditional processor-centric computing architecture and copper interconnects both reach their physical limit, the demands for higher-speed communication using silicon photonics are very high. Broadcom’s roadmap has switching chips going from 51.2 Tb/s this year to 102 Tb/s in 2024, and 205 Tb/s in 2026. This will be the big economic driver for silicon photonics enabling much higher capacity in the future.

**Q2: What are the major challenges of silicon photonics?**

A2: The major challenge is the integration of III–V materials into silicon CMOS which has progressed rapidly. Intel, Tower Semiconductor, and TSMC global are all working on this. There are remaining issues of high yields, high reliability, cost reduction, and fiber attach. The packaging of electronics and photonics together is a challenge, in particular, the temperature limits placed by the fiber attachment step. But again, progress is very rapid.

**Q3: Silicon is not an ideal platform for light emitters, but your group developed a unique approach to create active optical components on silicon and achieved mass production in just the last few years. How did you come up with this great idea?**

A3: Silicon is incredibly bad as a light emitter. Its internal quantum efficiency is about one part in a million, whereas a direct bandgap III–V material’s efficiency is essentially 100%. I knew from the beginning that we need to have a direct bandgap semiconductor, and I ignored most of the work on silicon, GeSn, and so forth. It was also clear that reliability would require high-quality materials. Defects are our big problems, particularly for lasers. Previously, we had developed bonded LEDs by putting GaAs on GaP back in the 1990s, and it was widely used in high-brightness LED by many manufacturers. So, it was natural to me to try bonding direct bandgap III–V to Si. And indeed, it worked very well. Our collaboration with Intel was essential to solving manufacturing issues and bringing it to high-volume production.

**Q4: After years of endeavor, what are the new challenges of light sources in silicon photonics? Your recent work also involves monolithic integration using quantum dot materials on silicon, how did you move into this research direction?**

A4: Heterogeneous integration has the advantage of being able to combine multiple materials together, bonded, and processed at one time. Conventional InP PICs have 5 or 6 regrowth steps, which is expensive, has problems, and limits the yield. With heterogeneous integration, one can bond the modulator, laser, and detector epi side by side at one time, and process them together. That’s always going to be the advantage of heterogeneous integration. But the cost of the substrate is not insignificant. The size of III–V substrates is far smaller than 300 mm. This drives our interest in monolithic integration. Fortunately, the work we did together has now resulted in high-quality, high-yield lasers on epitaxially grown 300 mm substrates. That’s very exciting, I initially never thought that would happen. It’s remarkable. A big issue in monolithic GaAs on Si devices was whether they are quantum wells or quantum dots. After 40 years of research, quantum well devices still have a lifetime of only 1000 h at high temperatures, but quantum dots have over 1,000,000 h of lifetime at high temperatures. The detailed science of dislocation flow is interesting and it’s important to solve these fundamental material issues. I’m continuing to be astounded by the differences. For the last 50 years, it has been difficult to make reliable GaAs pump lasers. The facet damage issues require delicate coating and processing. With silicon photonics, we can naturally get a reliable GaAs laser because we’ll never expose III–V facets. It’s been very lucky and fruitful.

**Figure:**
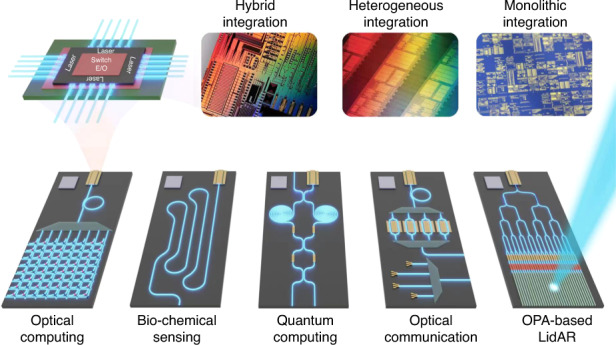
Different solutions to silicon lasers and their applications

**Q5: In addition to light sources, photodetectors and modulators are also important components in photonic integrated circuits. What poses the main challenges for high-speed and low-loss on-chip modulators? What about the noise of photodetectors for silicon photonics?**

A5: Typically, the main problem of modulators is microwave loss, especially at high speeds. For a conventional Lithium niobate modulator with a centimeter length, microwave loss is a big problem. When going to ring modulators with short lengths, people quickly achieved beyond 100 Gbit/s up to 180 Gbit/s. We’ll see the same thing with Mach-Zehnder modulators’. With the high confinement in silicon cladded by silicon dioxide, and III–V cladded by silicon dioxide, we can make the devices quite short and have very high performance. Similarly, for photodetectors, there are two really good solutions to the noise issue. One is that with gain on silicon, preamplified PIN detectors can be quite efficient: 10 dB better than just a PIN by itself. Secondly, silicon is an excellent avalanche material. We worked with Intel a decade ago and demonstrated III–V on silicon APDs with gain bandwidth products of 800 GHz. We will see that sort of technology become commercialized because silicon is such a superb avalanche material. Similarly, there are a lot of advances in germanium and silicon APDs. There is a lot of progress yet to be done there.

**Q6: Compared to integrated circuits whose scale has gone down to several nanometers, will the scale limit in silicon photonic imparts its potential?**

A6: There is certainly a big difference in scale. I doubt there will ever be a need for silicon photonics to use 3 nm lithography. 45 nm technology is sufficient to make high-performance, high-quality silicon photonics devices. That is good because working in an older foundry at a lower lithography level is much cheaper. By 3D bonding the PIC to electronics that may be 3 nm or beyond, it allows us to get the best of both worlds. So, I do not think it makes sense to integrate photonics and electronics onto the same wafer in the same process flow. It makes both process flows more expensive and longer. It makes much more sense to do 3D integration of the most advanced electronics with the most advanced photonics. Today, a 5 μm diameter ring modulator can achieve high-capacity interconnects compatible with the best processors. So, the sizes are not going to get that small for photonics devices.

**Q7: To process silicon photonics in CMOS foundries, how do people control the contamination issues, especially when heterogeneous integrated III–V materials are introduced?**

A7: I think what everyone does is to include the III–V materials, at the dirty end of the process, so they’re back in the copper end of the cycle. Most of these materials, indium, gallium, arsenic, and phosphorus, are already in the foundry. Therefore, operating in the dirty end of the foundry is not a big issue. For optical gyroscopes where low-loss optical waveguide is needed, high-temperature processing needs to be done prior to the introduction of III–V materials. The order of photonic integration steps does matter to solve that problem.

**Q8: Silicon photonics enables a wide range of applications, what’s your perspective on this?**

A8: Silicon photonics is ideal for any applications that have high volume needs to scale rapidly. Data centers are the biggest and the most immediate application. Telecommunications, as Acacia has demonstrated, is a second high-volume application, where the uniformity and superior performance from silicon processing really helps. The lithography in a 65 nm process is far better than in a typical InP foundry. Gratings and everything else can be directly written with high performance. A third general application is optical LIDARs, though the cost must be small, and the complexity might be large for an optical phase array. Scanning the beam in 2D requires matching to the electronic drivers to control the optical phase array. So, it becomes important to have both chips on the same substrate (silicon) for 3D integration. Optical gyroscopes are another example where the chips may be quite large. To get a sensitive rotation sensor, the chip is going to be at least a centimeter on the side. This is benefited from the large area substrates, high-volume, and low-cost processing of silicon. Today, the best gyroscopes are fiber optics based. They work very well, but they’re not integrated and are expensive. I think we can make better gyroscopes with silicon substrates and SiN waveguides.

**Figure:**
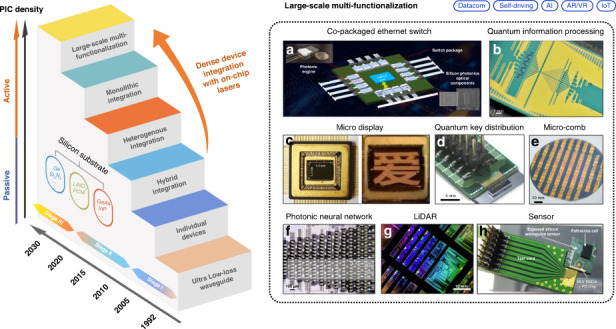
Silicon photonics enables a wide range of applications

**Q9: What chances can microcombs bring to silicon photonics?**

A9: The progress of microcombs is just phenomenal. If you look where we were a decade ago, and where we are today, it’s just astounding. A decade ago, combs were noisy and now it’s simple to make a single soliton and very quiet combs. I think the whole turnkey approach of generating solitons is the key. Every time you turn it on, you get a single soliton and low noise. Hundreds of lines can be generated, which are useful for applications including not only DWDM (dense wavelength division multiplexing), stable multi-wavelength sources, but also octave bandwidth generation for extremely quiet clocks. We’ve seen the ability to build an optical synthesizer where you can shift the frequency in steps of just 1 Hz across many THz of frequencies. I hope there will be a revolution analogous to what has happened when electronics developed synthesizers. Much better frequency control enabled a whole host of new applications. The ability to control laser frequencies to 1 Hz will open a lot of applications as well.

**Q10: How about quantum computing?**

A10: The whole artificial intelligence and machine learning field is quickly driving the need for extensive and more efficient computing. Optical computing is a natural way to do things like vector-matrix multiplications where you may not need 16-bit resolution, but you do need an answer efficiently. There are a whole host of companies using silicon photonics for optical computing, and I think it will have a big impact and work very well. Quantum computing is another step with a lot of inherent advantages. For instance, GaAs can be used to make entangled photons at high rates because of the large nonlinearity. This is very promising for future quantum applications and there’s a host of other ways to do it as well.

**Q11: What’s your perspective on other material platforms for photonic integration, LiNbO3, GaAs, etc.?**

A11: Thin film LiNbO_3_ on silicon has moved quickly. With thin film LiNbO_3_, the tight mode confinement helps to make high-speed modulators, comb generators, and a wild variety of devices. Lončar’s group at Harvard has really driven this with great success. Tightly confined modes work much better than the previous indiffused waveguides. GaAs is another good example. For GaAs resonators with waveguides that are tenths of microns high by eight-tenths of microns wide, you can get efficient second harmonic generation, photon entanglement, and comb generation. We’ve seen a comb generator with just 20 uW of power, which was inconceivable just ten years ago. There remain integration challenges. LiNbO_3_ has a very different coefficient of thermal expansion from silicon, which limits the processing when combined with the other process steps. GaAs materials have a closer range of thermal expansion coefficients with silicon, and you can certainly go to typically 400 or 500 °C. Oftentimes, when you include LiNbO_3_ with the rest of the silicon photonics, you’re limited to 200 or 250 °C. Those can be solved in a back-end process.

**Q12: What would be your next research focus?**

A12: Just to expand the range of applications. The use of silicon photonics in data centers is well-established. Intel has a billion-dollar business doing that today and many other companies are also actively involved. With the foundry efforts from Tower, TSMC, Global Foundries, and AIM, there will be tons of opportunities open for other applications as well, LIDARs, gyroscopes, and spectroscopy, for example. Dual comb spectroscopy is one really exciting advance that will allow us to sense a variety of pollutants, greenhouse gases, and medical sensing of things in our blood and so forth. That is what I am excited about in the future. In addition, most silicon photonics is still in the infrared, but going into the visible is important. The scope of silicon photonics expands to those using silicon substrate and silicon processing. The waveguides are not limited to silicon, but can cover a wide range of materials, including LiNbO_3_ waveguides, compound semiconductors, CSOI, SiN, etc. Nexus photonics is a company integrating lasers with silicon nitride waveguides. Using SiN waveguides, they make 980 nm tunable lasers operate up to 185 °C, which is phenomenal. I also expect to see a lot of atomic clock applications, display applications, headsup displays on glasses and contact lenses, and so forth, with the SiN-based silicon photonics.

**Q13: Beyond a leading scientist, you are also known for commercializing several products and incubating several corporations successfully. What are the key factors from research to commercialization?**

A13: The biggest key factor is certainly hiring smart people. I have been very lucky to have a lot of smart students and postdocs that left the group and started companies. I am glad I was able to help them. It makes the research real for students if they achieve success in the university lab and later are able to commercialize it. It’s very satisfying for all of us. In particular, Alex Fang with Aurrion, Jon Geske with Aerius Photonics, Tin Komljenovic with Nexus, and Alan Liu with Quintessent have successfully applied research they’ve done for real products and hopefully changing the world.

**Q14: The commercialization of silicon photonics has attracted enormous venture capital. Can the development of silicon photonics keep pace with the expansion of its startup companies?**

A14: Historically, hardware companies required a lot of money to be successful because you must build a fab. If you look at Infinera, the first thing they did was to build a clean room which was very expensive and took a long time. Now we have the capability of accessing well-established, well-funded advanced foundries. Those foundries are paid for by electronics but can now be used for photonics. This allows startup companies to get established and get a product out without much capital, and then to scale rapidly. Intel went from making its first 100 GB transceiver to making 3 million transceivers a year in just 3 years. That’s the strength of consumable electronics and conventional CMOS foundries.

**Q15: What was the influence of your work experience at Bell Lab on your choice of silicon photonics as a research focus at UCSB?**

A15: I was lucky to go to Bell Labs at the very beginning of fiber optics. I arrived in 1982, and the first fiber-optic systems were just being deployed with relatively modest bit rates, one hundred Mbit/s sorts of systems. The problems were very clear. We had to move to longer wavelengths, to 1.3 and 1.55 μm, and move to single-frequency lasers. There were very clear research directions, and you could go down the hallway and find an expert in whatever you needed, whether it was about fibers, epitaxy or processing. We learned to work together and moved rapidly to the first 2, 4, 8, and 16 Gb/s systems, and then the whole development of DWDM, and the optically amplified systems. When I went to UCSB, that was all established, and the Internet became widely available. We first worked on VCSELs, particularly using bonding to make long-wavelength VCSELs, LEDs, and mode-locked lasers for comb sources. Then, when silicon photonics started exploding, the need for a laser on silicon was obvious. Every photonic integrated circuit had to have an integrated laser. It really did not make sense for me to do anything else. You can always have a fiber-coupled laser, but that limits the complexity with hundreds of sources. Electronics can succeed because you have cheap gain everywhere and complex integrated circuits can’t be made without gain. The same thing is true for fiber optics. It was the development of the EDFA that enabled low-cost, high-capacity fiber-optic systems, which allowed the explosion of fiber optics to occur. The same thing is true for photonic integrated circuits. You need to have gain on chips, whether it’s the laser or just the amplifier, to make bigger and more complex chips. We are just at the very beginning of that field, and we will see much better performance in the future.

**Q16: Why did you choose academia as your career path?**

A16: I really enjoy teaching. I enjoy seeing students become successful. Some arrive without even knowing how to use an oscilloscope, and they graduate as a world leader in their field. That is something they should be very proud of, and I am also very proud of what they have accomplished. Being able to help them succeed, I feel very happy, and I still do. Early on, I was quite involved in running the startups that came out of my group, Terabit or Calient, for example. But now, students are the leading people in running the company and my role is just to help them become successful. I’m very proud of the many students, who have gone on to become tenured professors, and do a better job at teaching and research than I do.

**Figure:**
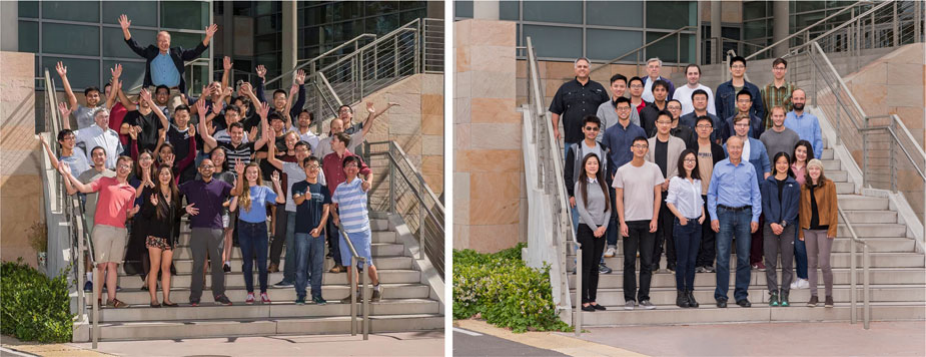
Prof. Bowers and his students

**Q17: What part of your career makes you most excited?**

A17: I think it is changing the world. If we can make photonics on silicon rather than on InP or GaAs, if we can solve medical problems, and make very efficient quantum computers using silicon photonics, that will make me very happy. Changing the world is always a team effort and a team sport. The big roles are typically played by others, like Alex Fang or others that lead a team at a company. I am happy to support the effort.

**Q18: You have supervised more than 80 PhD students and postdocs and most of them still work in integrated photonics. How did you inspire and unite your students to work in this field?**

A18: This is a great field to be in and it’s still exploding. Different fields explode at different times, then it sorts of saturates, and then becomes very mature. Telecommunications is becoming mature. A lot of great work at Acacia led to advanced silicon photonics coherent systems and demonstrate the quality of what you can do in a CMOS foundry. That is an important, yet a mature field. Data centers are still exploding with a lot of innovation and we are still in the early stage. In the other areas, optical computing, quantum computing, sensing, etc., we are just at the very beginning. There will be many students making very sophisticated devices in these areas, far beyond what we see today. I think it is important to pick a field that is expanding. The other big advantage of silicon photonics lies in its big economic driver, namely, the need to marry electronics and photonics together. We can make better photonics because we have 3D-integrated electronics to drive it intimately. We can make better electronics because we have photonics to do the optical interconnects. That’s a big economic driver and everything else can succeed and flow from that.

**Q19: Silicon photonic is now the key technology for next-generation data communication. But 20 years back, how did you persuade your students to work in this new field?**

A19: Like Wayne Gretzky says, go to where the puck is going, not to where it is. When I first came to UCSB, we had a bunch of big laser systems, but we rapidly moved to semiconductors and fiber optics. Photonic integrated circuits are cost-effective and can scale rapidly. That’s the direction I have always taken, and I encouraged my students to do the same.

**Q20: You are the role model for many researchers. Who was your role model when you started your career?**

A20: My advisor, Gordon Kino was my biggest role model. He was a brilliant man, originally a mathematician, and really led the theory of what we were doing in the lab. From him, I learned to always combine experiments and theory together. My recent role models include people like Rod Alferness, who has been the dean at UCSB and was the department head and chief scientist at Bell Lab before that. He is sort of the premier leader of research, always been able to encourage researchers and give them good advice and steer them in the right direction with the resources they need. That has also been my goal. If I can make sure the students have the resources to do the research they want to do, and not be limited by the equipment, they can be more self-motivated and make a difference.

**Q21: What motivated you to pursue a PhD in the early days? What kind of advice would you like to share with students who were just starting their academic career?**

A21: Certainly, one thing is just to get in the lab and do real research. When I was an undergraduate, I was fortunate to work with the high-energy physics group that Marvin Marshak led at the University of Minnesota, with experiments at Argonne National Lab and Fermi Lab. It was exciting. I was a physics major, and I was convinced that’s what I wanted to do. But it also became clear that not many high-energy physics graduates became tenured professors, probably one in a hundred, I suspect. So that drove me to shift to solid-state physics for my PhD. Almost everyone I knew stayed in the field of solid-state physics later on, whereas too many of the high-energy physicists became computer scientists after ten years. I went to graduate school in the late 70 s, and that’s when fiber optics was just beginning. It was clear that it was a good field to go into. My advice to students is to find your passion and pursue your thesis passionately. Grad school is a great opportunity to do great research.

**Q22: You have taken a lot of responsibilities in both the university and the industry business. How did you manage to multi-task?**

A22: Multi-tasking is required for all of us. Prioritizing work and being organized are the key. I often tell graduate students that getting a thesis is frustrating. You must have reasonable expectations that it’s going to be hard work. When you hit that low point that nothing is working, when you’re failing and everyone else seems to be successful, you must continue. You can not just fail around and jump to some other area, you should stick with it. So be prepared there’ll be great, exciting days, but also be prepared that there might be very low days when nothing’s working. You must be determined and pursue it despite difficulties. And indeed, the harder the problem, the more prominent your success will be.

**Q23: How did you make a balance between work and life?**

A23: I think it is important to get exercise. It is hard when there is a lot of work demands in your job and family demands in your life. For me, I get up most days at about 6:00 am and I bike for the first hour. Regular exercises help me manage stress and stay healthy. Once you have kids, engaging in their activities, whether it’s soccer or something else, is important.

**Q24: What kind of exercise do you most suggest?**

A24: At Bell Labs, we had a group of about ten of us who went running every day at lunchtime, that was great. My knees are not what they used to be, so I can’t run long distances anymore. Biking, skiing, and playing pickleball is much more of my preference now.

**Figure:**
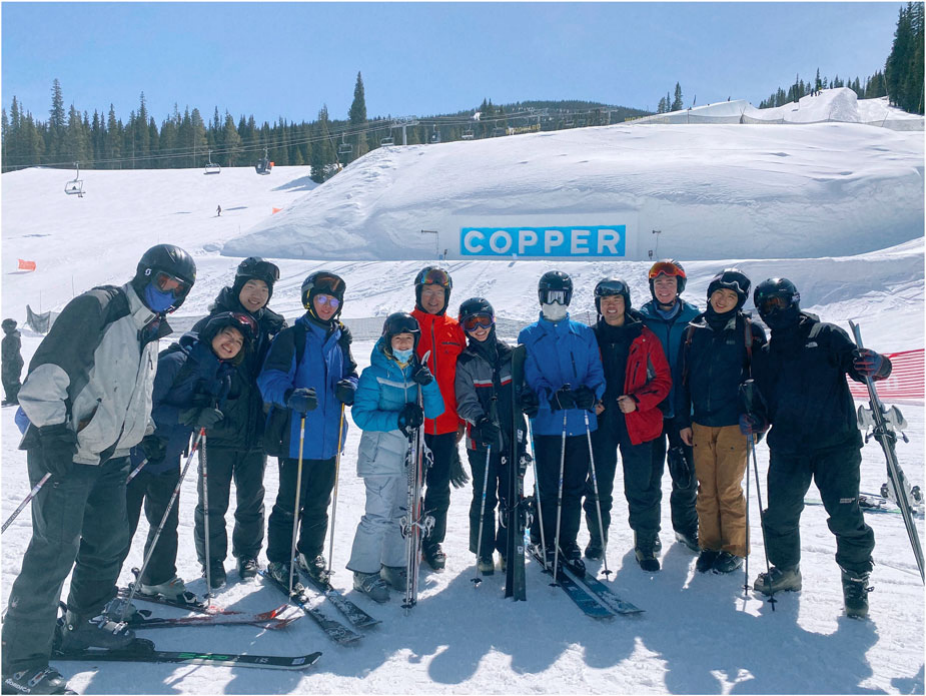
Prof. Bowers and his group members on a ski trip in Colorado

**Q25: The past decades witnessed a boom in journals, and we are very lucky to have your support for our three journals, LSA, eLight, and Light: Advanced Manufacturing. What do you think constitutes a good journal?**

A25: Well, I think LSA, eLight, and LAM are very high-quality journals, and I have been lucky to publish in all three. I think high standards and rapid handling of the processing of the manuscripts are key, and the three journals do both very well.

## Supplementary information

Supplementary Video

